# Initial real-world data on catheter ablation in patients with persistent atrial fibrillation using the novel lattice-tip focal pulsed-field ablation catheter

**DOI:** 10.1093/europace/euae129

**Published:** 2024-06-25

**Authors:** Shota Tohoku, Stefano Bordignon, David Schaack, Jun Hirokami, Lukas Urbanek, Andrea Urbani, Joseph Kheir, Boris Schmidt, Kyoung-Ryul Julian Chun

**Affiliations:** Cardioangiologisches Centrum Bethanien Med. Klinik III, Markuskrankenhaus, Department of Cardiology, Wilhelm-Epstein Str. 4, 60431 Frankfurt, Germany; Klinik für Rhythmologie, Universitätsklinikum Schleswig-Holstein der Universität zu Lübeck, Ratzeburger Allee 16023538 Lübeck, Germany; Cardioangiologisches Centrum Bethanien Med. Klinik III, Markuskrankenhaus, Department of Cardiology, Wilhelm-Epstein Str. 4, 60431 Frankfurt, Germany; Cardioangiologisches Centrum Bethanien Med. Klinik III, Markuskrankenhaus, Department of Cardiology, Wilhelm-Epstein Str. 4, 60431 Frankfurt, Germany; Cardioangiologisches Centrum Bethanien Med. Klinik III, Markuskrankenhaus, Department of Cardiology, Wilhelm-Epstein Str. 4, 60431 Frankfurt, Germany; Cardioangiologisches Centrum Bethanien Med. Klinik III, Markuskrankenhaus, Department of Cardiology, Wilhelm-Epstein Str. 4, 60431 Frankfurt, Germany; Cardioangiologisches Centrum Bethanien Med. Klinik III, Markuskrankenhaus, Department of Cardiology, Wilhelm-Epstein Str. 4, 60431 Frankfurt, Germany; Cardioangiologisches Centrum Bethanien Med. Klinik III, Markuskrankenhaus, Department of Cardiology, Wilhelm-Epstein Str. 4, 60431 Frankfurt, Germany; Cardioangiologisches Centrum Bethanien Med. Klinik III, Markuskrankenhaus, Department of Cardiology, Wilhelm-Epstein Str. 4, 60431 Frankfurt, Germany; Universitätsklinikum Frankfurt, Medizinische Klinik 3- Klinik für Kardiologie, Frankfurt, Germany; Cardioangiologisches Centrum Bethanien Med. Klinik III, Markuskrankenhaus, Department of Cardiology, Wilhelm-Epstein Str. 4, 60431 Frankfurt, Germany; Klinik für Rhythmologie, Universitätsklinikum Schleswig-Holstein der Universität zu Lübeck, Ratzeburger Allee 16023538 Lübeck, Germany

**Keywords:** Persistent atrial fibrillation, Pulsed-field ablation, Lattice-tip catheter

## Abstract

**Aims:**

Technological advancements have contributed to the enhanced precision and lesion flexibility in pulsed-field ablation (PFA) by integrating a three-dimensional mapping system combined with a point-by-point ablation strategy. Data regarding the feasibility of this technology remain limited to some clinical trials. This study aims to elucidate initial real-world data on catheter ablation utilizing a lattice-tip focal PFA/radiofrequency ablation (RFA) catheter in patients with persistent atrial fibrillation (AF).

**Methods and results:**

Consecutive patients who underwent catheter ablation for persistent AF via the lattice-tip PFA/RFA catheter were enrolled. We evaluated acute procedural data including periprocedural data as well as the clinical follow-up within a 90-day blanking period. In total, 28 patients with persistent AF underwent AF ablation either under general anaesthesia (*n* = 6) or deep sedation (*n* = 22). In all patients, pulmonary vein isolation was successfully achieved. Additional linear ablations were conducted in 21 patients (78%) with a combination of successful anterior line (*n* = 13, 46%) and roof line (*n* = 19, 68%). The median procedural and fluoroscopic times were 97 (interquartile range, IQR: 80–114) min and 8.5 (IQR: 7.2–9.5) min, respectively. A total of 27 patients (96%) were interviewed during the follow-up within the blanking period, and early recurrent AF was documented in four patients (15%) including one case of recurrent AF during the hospital stay. Neither major nor minor procedural complication occurred.

**Conclusion:**

In terms of real-world data, our data confirmed AF ablation feasibility utilizing the lattice-tip focal PFA/RFA catheter in patients with persistent AF.

## Graphical Abstract

**Graphical Abstract:**
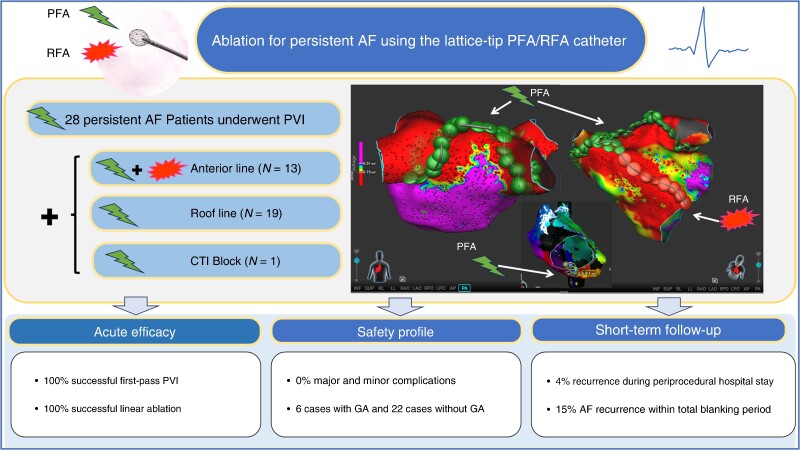

**What’s new?**

- This is the initial real-world report on treating patients with persistent atrial fibrillation using the novel lattice-tip pulsed-field ablation (PFA)/radiofrequency current ablation (RFA) catheter.
- All procedures succeeded irrespective of utilization of general anaesthesia.
- 100% complete successful PVI with first-pass isolation and additional linear ablation solely using the lattice-tip PFA/RFA catheter was achieved.
- No recorded periprocedural complication indicated a potential remarkable safety profile
- Recurrent atrial fibrillation was detected in 15% of patients within the 90-day blanking period

## Introduction

Achieving sustained pulmonary vein (PV) isolation (PVI) remains a fundamental and crucial challenge in atrial fibrillation (AF) ablation.^1^ To date, pulsed-field ablation (PFA) has tried to open up a new frontier in AF ablation as a myocardium-specific non-thermal ablation technique, with efficacy and optimal safety profiles for clinical applications.^2–6^

In Europe, the first PFA device officially approved showed high single-procedure success rates with an excellent safety profile and short procedure time not only in preclinical studies but also in real-world data.^3–5^ However, patients with persistent AF exhibited significant higher rates of recurrence compared to those with paroxysmal AF despite a high rate of durable antral PVI,^5,7^ which still requires the need for a further step in the ablation strategy for patients with advanced-staged AF.

Although the clinical benefit of empirical substrate modification beyond PVI could not reach a concensus,^8–11^ some individualized ablation strategies showed improved outcomes.^12,13^ An influence of target selection via high-resolution mapping and improved technical aspects of energy delivery could play a role in substrate modification. Recently, in Europe, a novel unique PFA system has been clinically available which comprises (i): a 9 mm compressive lattice-tip catheter capable of delivering energy in a point-by-point strategy as the core of the whole system, (ii): a generator that allows a alternate energy output between the PFA and radiofrequency ablation, and (iii): integration of a proprietary high-resolution three-dimensional mapping system enhancing preciseness of anatomical and electrophysiological understanding. Combining these characteristics could facilitate in identifying the targeted substrate and to form a better lesion, which suitably contributes to ‘beyond PVI’, therefore, patients with persistent AF would benefit from these novelties. After the first-in-human trials in each energy source,^2,6^ the feasibility of this technology was shown in a larger-scale multicentre clinical trial.^3^ As the next step, this study aimed to confirm the feasibility of these trials in terms of real-world data after commercialization through the elucidation of our initial clinical experience via the lattice-tip PFA/RFA catheter in patients with persistent AF.

## Methods

All patients provided written informed consent before undergoing the procedure. The study was approved by the Institutional Review Board of Cardiovascular Centrum Bethanien and complied with the Declaration of Helsinki.

### Study protocol

This is an observational single-centre study. Acute procedural data and short-term clinical follow-up were evaluated in patients with persistent AF following initial ablation using the lattice-tip PFA/RFA catheter. Early recurrence was defined as documented AF/atrial tachycardia (AT) lasting more than 30 s within a 90-day blanking period after the procedure.^14^

### Patient population

Consecutive patients with symptomatic persistent AF refractory to the treatment of at least one antiarrhythmic drug (AAD) including beta-blockers (Class I–III) were eligible. The exclusion criteria included patients with previous PVI attempts or were ineligible for treatment with oral anticoagulation.

### Pre-ablation protocol

Anticoagulation was continued until the morning of the procedure and resumed in the evening after the intervention. To exclude intracardiac thrombi, transoesophageal echocardiography was routinely conducted at a maximum of 30 days prior to the procedure. Preprocedural imaging to evaluate the PV anatomy was not required.

### Investigational device

The detailed system of ‘Affera^™^ Mapping and Ablation System’ (Medtronic, Minneapolis, MN, USA) has been previously described.^2,3,6^ Briefly, an 8 Fr bidirectional catheter (Sphere-9^™^ Catheter) with an expandable 9 mm lattice-tip configuration equipped with a total of nine small thermocouple functioning as mini-electrodes on its surface is the key component of this system. Furthermore, a single indifferent electrode and two ring electrodes are on the distal catheter shaft inside the lattice part. The catheter is coupled with a proprietary generator designed for PFA (HexaPULSE^™^, Medtronic) and RFA (HexaGEN^™^, Medtronic) to ascertain that physicians are able to deliver wide-area focal ablation lesions of choice between PFA and RFA, according to the patient and procedure needs. The system also includes its proprietary three-dimensional electroanatomic mapping system based on magnetic localization (Prism-1 Mapping^™^ software, Medtronic).

Every application of PFA was delivered with a predefined optimal energy setting (PULSE3),^3,6^ that is, a preclinically defined optimized biphasic monopolar waveform in 4 s using saline irrigation. Radiofrequency ablation application was delivered under temperature-control in 5 s with saline irrigation with a targeted surface temperature of 73–75°C. In both energies, a targeted centre-to-centre distance between adjacent lesions was set as 5–6 mm.

### Ablation protocol

Procedures were conducted via five different first operators with more than a 5-year clinical experience in the field of invasive cardiac electrophysiology. General anaesthesia (GA) was used for the initial consecutive patient including the administration of muscle relaxants. For subsequent patients, procedures were conducted under intravenous sedation using propofol, midazolam, and fentanyl, in adherence with the recommendations of a German positional paper.^15^ Intravenous heparin was administered to maintain an activated clotting time of ≥300 s prior to placing the femoral venous access under ultrasound echo guidance. If the electric cardioversion was successful, sinus rhythm was restored. A 6 Fr decapolar diagnostic catheter (Inquiry 6 Fr, Abbott) was positioned in the coronary sinus. Double transseptal punctures were conducted using an 8 Fr sheath (SL1; Abbott) and a Brockenbrough needle under fluoroscopic guidance. Selective PV angiographies following single a transseptal puncture were conducted with a 7-Fr multipurpose catheter in a standard angulation (right anterior oblique 30°/left anterior oblique 40°).

An ablation catheter and a spiral diagnostic catheter were delivered via the transseptal sheath in the left atrium (LA). Using an ablation catheter, a three-dimensional voltage map was constructed. For the lateral PVs, a repeat three-dimensional map was built to define the border between the PV and left atrial appendage (LAA) to prevent accidental energy delivery around the LAA base.

**Figure 1:**
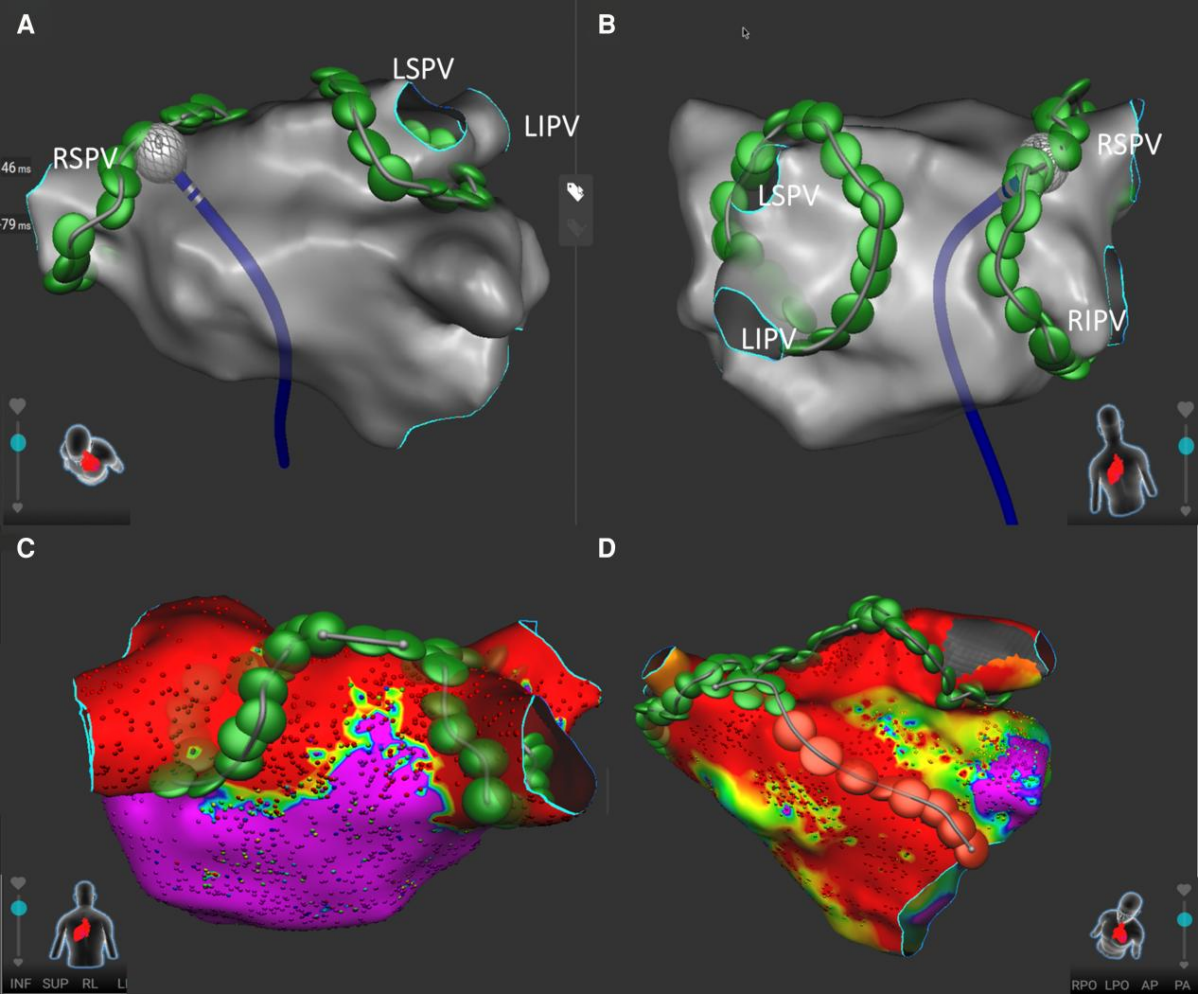
Representative three-dimensional anatomical maps of left atrium after pulmonary vein isolation (*A*, *B*) and substrate modification with a roof line ablation (*C*) and an anterior mitral isthmus line ablation (*D*) in anterior–posterior and posterior–anterior angulations. LIPV: left inferior pulmonary vein; LSPV: left superior pulmonary vein; RIPV: right inferior pulmonary vein; RSPV: right superior pulmonary vein.

Ipsilateral PVs were circumferentially isolated using the PFA (Figure *1A* and *B*). After confirming the entrance block of the PVs using the spiral diagnostic catheter, the potentials for latent PV reconnection were reassessed after a waiting time during linear ablation or intravenous adenosine challenge.

If the following findings were detected, a substrate modification with linear ablations (Figure *1C* and *D*) was conducted, (i); low-voltage area, that is, the tissue bipolar voltage <0.5 mV, was detected, (ii); the conduction time from the beginning of the *P* wave to the LAA > 100 ms as a marker indicating the conduction delay of the Bachmann bundle and/or the anterior wall of the LA.^16^

**Figure 2:**
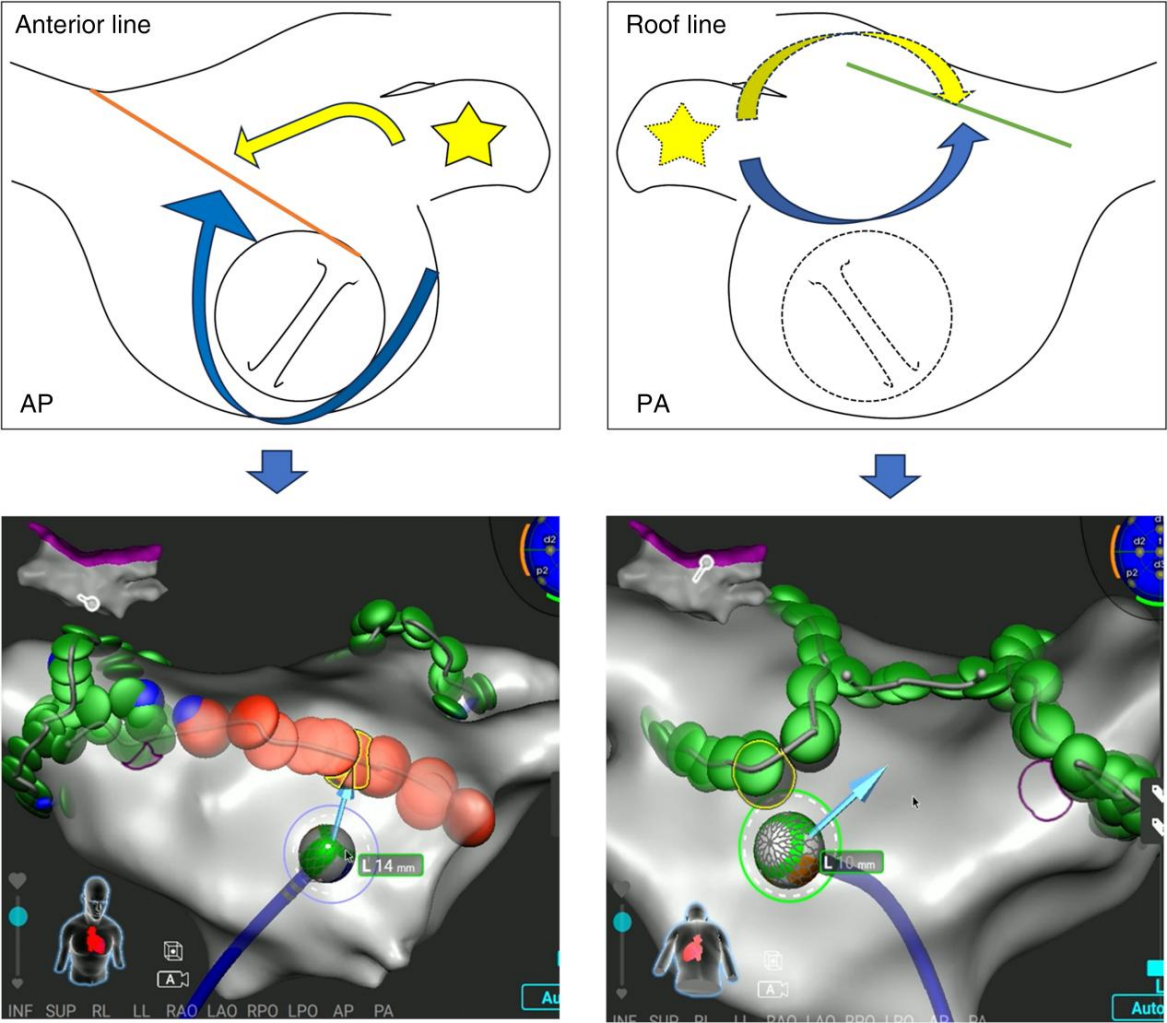
Real-time vector function indicating blocks of an anterior mitral isthmus line (left) and a roof line (right). Stimulation is applied from the left atrial appendage (star). The pacing stimulus from the left atrial appendage travels from both directions towards the ablation line, that indicates conductions in opposite directions colliding with each other on the lines.

Pulsed-field ablation was exclusively used based on clinical trial data for the roof line ablation and cavotricuspid isthmus (CTI) block.^3,6^ Anterior mitral isthmus line was conducted combining the RFA between the mitral anulus and the anteroseptal site of the right superior PV antrum, and PFA on the rest of the line through toggling the energy source. To perform a differential pacing manoeuvre to verify the bidirectional block of linear ablations, the spiral catheter was placed in the LAA. Real-time vector function providing a direction of local activation was also utilized (*Figure 2*).

In case of an incomplete block of linear ablations and PV reconnection, additional ablation with RFA was employed on breakthrough sites.

### Follow-up

Class Ⅰ or Ⅲ AAD administration was discontinued immediately after the procedure. A 90-day blanking period after the procedure was applied. A follow-up analysis was mainly conducted via telephone interview. Patients experiencing early recurrent atrial tachyarrhythmia within the blanking period were encouraged to continue AAD until the end of the period.

A recurrence was defined as any documented atrial tachyarrhythmia episode lasting >30 s.

### Statistical analysis

In light of its exploratory nature, this study employs descriptive statistical analysis to demonstrate outcomes and safety parameters associated with the novel intervention. Given the absence of a predefined hypothesis testing framework or power calculation, the analysis primarily focuses on characterizing the data. Continuous variables are presented as either mean with standard deviation or median with interquartile range (IQR). Meanwhile, categorical variables are delineated by counts and corresponding percentages, providing a comprehensive overview of the study cohort. Analyses were conducted using JMP version 11.0 (SAS Institute, Cary, NC, USA).

## Results

### Patient backgrounds

A total of 32 procedures were conducted using the lattice-tip PFA/RFA catheter between August and December 2023. Four patients were excluded from the analysis due to the history of previous ablation procedures. In total, 28 patients with persistent AF including two patients with long-standing persistent AF were included in the study. Patient characteristics are summarized in *Table 1*. Patients have a median age of 72 (IQR: 62–79) years old, and the vast majority of patients were overweight [median body mass index (BMI), 28.5 (IQR: 26.3–31)]. Twelve patients had a history of electrocardioversion before the procedure.

**Table 1:** Patient characteristics

Patient characteristics	*n* = 28
Age (years)	72 (IQR: 62–79)
Gender (Female), %, *n*	32% (9)
BMI	28.5 (IQR: 26.3–31)
Hypertension, %, *n*	75% (21)
Coronary artery disease, %, *n*	18% (5)
Heart failure, %, *n*	3.5% (1)
Diabetes mellitus, %, *n*	14% (4)
History of stroke, %, *n*	11% (3)
Left atrial diameter (mm)	41 ± 5
Ejection fraction (%)	57 ± 7
Duration of AF after the first diagnosis, (month)	19 (IQR: 9–41)
Longest episode prior to ablation, (month)	4 (IQR: 3–7)
Current anti arrhythmic drug, %, *n*	22 (79%)
Class Ⅰ, %, *n*	7% (2)
Class Ⅱ, %, *n*	79% (22)
Class Ⅲ, %, *n*	0% (0)

BMI, body mass index; IQR, interquartile range.

### Procedural data

#### General anaesthesia or deep sedation

The first six patients underwent procedures under GA. The subsequent patients were deeply sedated. A single case required mechanical ventilation using a laryngeal mask due to severe obstructive sleep apnoea syndrome in a patient with a BMI of 41.

#### Pulmonary vein isolation

**Figure 3:**
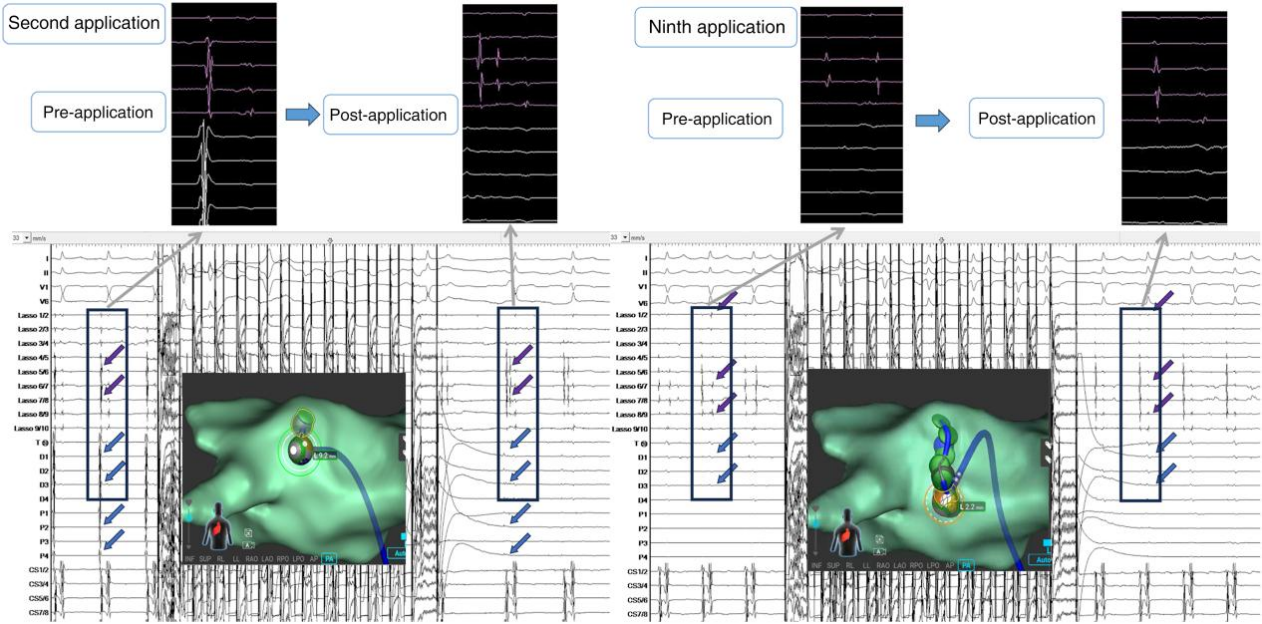
A case of real-time isolation of PV. The PV potential was delayed after the second application (left), then disappeared after the ninth application (right). Arrows indicate recorded PV potentials on spiral mapping and ablation catheters.

A total of 111 PVs including 1 left common PV were identified. All PVs were isolated solely using PFA applications. First-pass isolation, that is, successful PVI after the first circumferential lesion set on the PV antrum, was achieved in all PVs. Real-time isolation during ablation was noted in 47/55 (86%) ipsilateral PV side in which the disappearance of PV potentials was noted after ablation around the PV antrum halfway around (*Figure 3*). In other PVs, a spiral diagnostic catheter was placed in the contralateral PV to facilitate catheter manipulation during ablation. In 20 patients, adenosine challenge was conducted. None of the PVs showed acute reconnection. In total, the number of applications needed for PVI was 93 (IQR: 63–107), [septal PVs: 53 (IQR: 40–62) and lateral PVs: 47 (IQR: 39–54)].

#### Beyond pulmonary vein isolation

In three cases, peri-mitral isthmus-dependent AT, atrial flutter, and typical atrioventricular nodal re-entry tachycardia were induced coincidentally and were thus treated during the same sessions.

The anterior mitral isthmus line and roof line ablation were additionally performed in 13 (46%) and 19 (68%) patients as substrate modification. Radiofrequency ablation application was exclusively used for the anterior line. Data for each linear segment was summarized in *Table 2*. The aforementioned AT was successfully treated with an anterior line. For the patient with an atrial flutter, a CTI block was conducted terminating the tachycardia with 18 PFA applications. Nitroglycerine was administered prior to CTI ablation and no change was noted on the ST-segment in the 12-lead electrocardiogram (ECG) of this patient. For the patient with typical atrioventricular nodal re-entry tachycardia, a slow-pathway ablation was conducted via an additional conventional RFA catheter. Procedural characteristics are summarized in *Table 2*.

**Table 2:** Procedural data

Procedural data	28 patients, 110 PVs
Procedure time (min)	97 (IQR: 80–114)
LA dwell time (min)	72 (IQR: 60–94)
Fluoroscopic time (min)	8.5 (IQR: 7.2–9.5)
First-pass isolation, %, *n*	100% (110/110)
Real-time isolation (ipsilateral side), %, *n*	86% (47/55)
Additional linear ablations, %, *n*	75% (21/28)
Anterior mitral isthmus line, %, *n*	46% (13/28)
Roof line, %, *n*	68% (19/28)
CTI ablation, %, *n*	3.5% (1/28)
Additional ablation (AVNRT), %, *n*	3.5% (1/28)
Number of applications for PVI (total PVs), %, *n*	93 (IQR: 63–107)
Number of applications for PVI (septal PVs), %, *n*	53 (IQR: 40–62)
Number of applications for PVI (lateral PVs), %, *n*	47 (IQR: 39–54)
Number of applications for anterior mitral isthmus line (PFA)	7 (IQR: 5–10)
Number of applications for anterior mitral isthmus line (RFA)	11 (IQR: 8–13)
Number of applications for roof line (PFA)	8 (IQR: 7–11)
Number of applications for CTI ablation (PFA)	18
Transpired ablation time, (min)^a^	
PV Isolation, (min)	26 (IQR: 20–38)
Anterior mitral isthmus line, (min)	7 (IQR: 5–10)
Roof line, (min)	3 (IQR: 2–4)
CTI ablation, (min)	2
Mapping time, min,	17 (IQR: 14–21)
Number of points in three-dimensional map, *n*	3442 (IQR: 3142–4436)
Visualized left atrium volume, cc	146 (IQR: 126–174)

PVs, pulmonary veins; PVI, pulmonary vein isolation; LA, left atrium; CTI, cavotricuspid AVNRT, atrioventricular nodal reentry tachycardia; CTI, cavotricuspid isthmus; IQR, interquartile range; LA, left atrium; PFA, pulsed-field ablation; PV, pulmonary vein; PVI, pulmonary vein isolation; RFA, radiofrequency current ablation.

^a^Defined as the time transpiring from first to last lesion of the particular lesion set.

#### Three-dimensional mapping system

All procedures were conducted under the three-dimensional mapping guidance. The number of electrogram taken on the three-dimensional map was 3442 (IQR: 3142–4436) with a median atrial volume of 146 (IQR: 126–174) cc. The total procedure and fluoroscopy times recorded 97 (IQR: 80–114) min and 8.5 (IQR: 7.2–9.5) min, respectively.

#### Periprocedural complications

Neither critical complications, that is, symptomatic cerebral stroke, cardiac tamponade, or oesophageal fistula nor minor complication occurred (*Table 3*).

**Table 3:** Safety profile

Safety profile	28 procedures
Total complications, %, *n*	0% (0/28)
Cerebral vascular event, %, *n*	0% (0/28)
Cardiac tamponade, %, *n*	0% (0/28)
Gastrointestinal symptom, %, *n*	0% (0/28)
Transient phrenic nerve palsy, %, *n*	0% (0/28)
Vascular access complication, %, *n*	0% (0/28)

#### Follow-up

During hospitalization, sinus rhythm was restored in 27 patients. One patient presented with recurrent AF on the following day after ablation, which spontaneously converted to sinus rhythm. Until end of the blanking period, this patient was treated with amiodarone. Three months after treatment, seven individuals attended follow-up at our outpatient clinic while the remaining 21 were primarily followed-up through telephone surveys, with 20 respondents participating in the survey. Of these, 14 patients visited the family doctors. In total, follow-up during 90-days blanking period was completed in 27 patients (96%). Holter-ECG was conducted in eight patients. Recurrent AF was documented in 4/27 patients (15%). The rest of the three patients with recurrent AF were treated with beta-blocker administration. Two patients were still symptomatic after ablation (7%) with documentation of symptomatic atrial premature beats at Holter recording.

## Discussion

The main findings of the study are as follows: (i) AF ablation in patients with persistent AF using the lattice-tip PFA/RFA catheter succeeded favourably irrespective of the need for GA. (ii) A 100% complete successful PVI with the first-pass isolation, including an additional linear ablation solely using the lattice-tip PFA/RFA catheter, was achieved. (iii) The first evidence of a remarkable safety profile with no complication was recorded. (iv) A 15% recurrence within the 90-day blanking period was also noted.

### Ablation workflow without general anaesthesia

**Figure 4:**
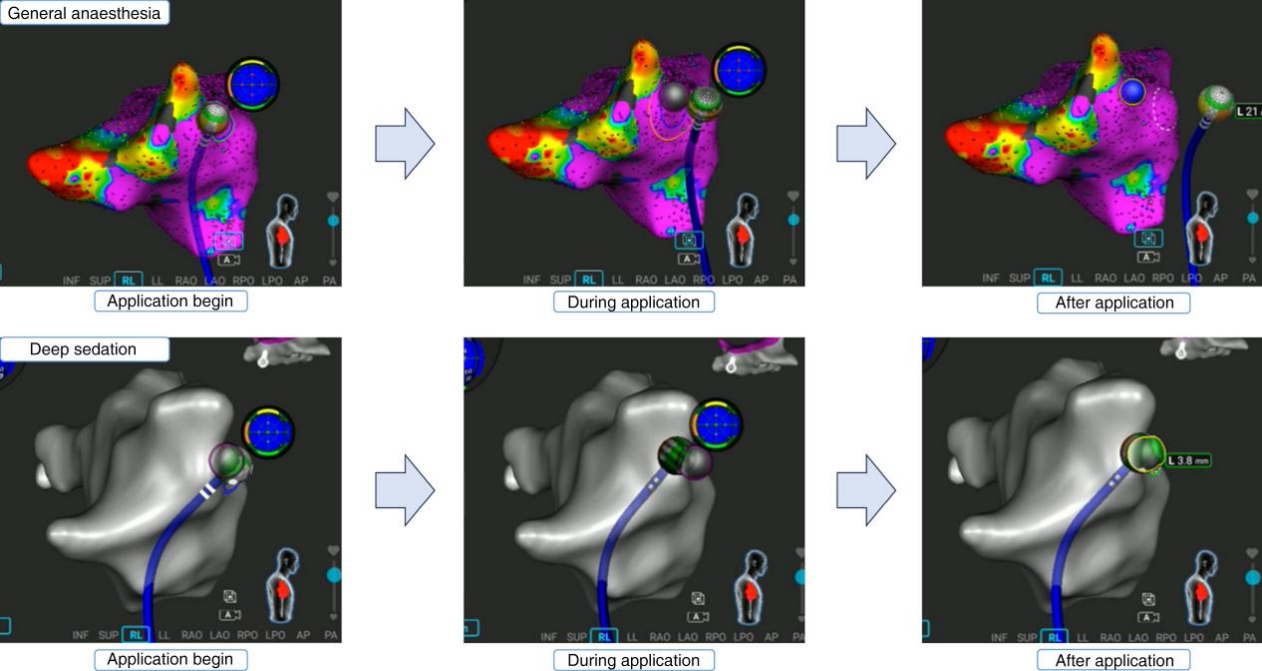
Comparison of the catheter stability on the three-dimensional mapping system during an application between deep sedation (upper) and general anaesthesia (below). The catheter position dislocated forward due to a diaphragm contraction and subsequent coughing in the procedure under deep sedation.

Our study preliminary reveals the feasibility of procedures using the unipolar PFA catheter performed under deep sedation. Only a single case required mechanical ventilate support due to the cause independent of the ablation system. Despite a transient macroscopic dislodgement of the catheter due to diaphragm contraction (*Figure 4*; Supplementary material online, *Video S1*), first-pass isolation was achieved in all PVs. Moreover, no map shift was observed. Further large-scale data comparing chronic lesion durability are warranted to clarify the need of GA during point-by-point PFA procedures.

### Pulmonary vein isolation with PFA

**Figure 5:**
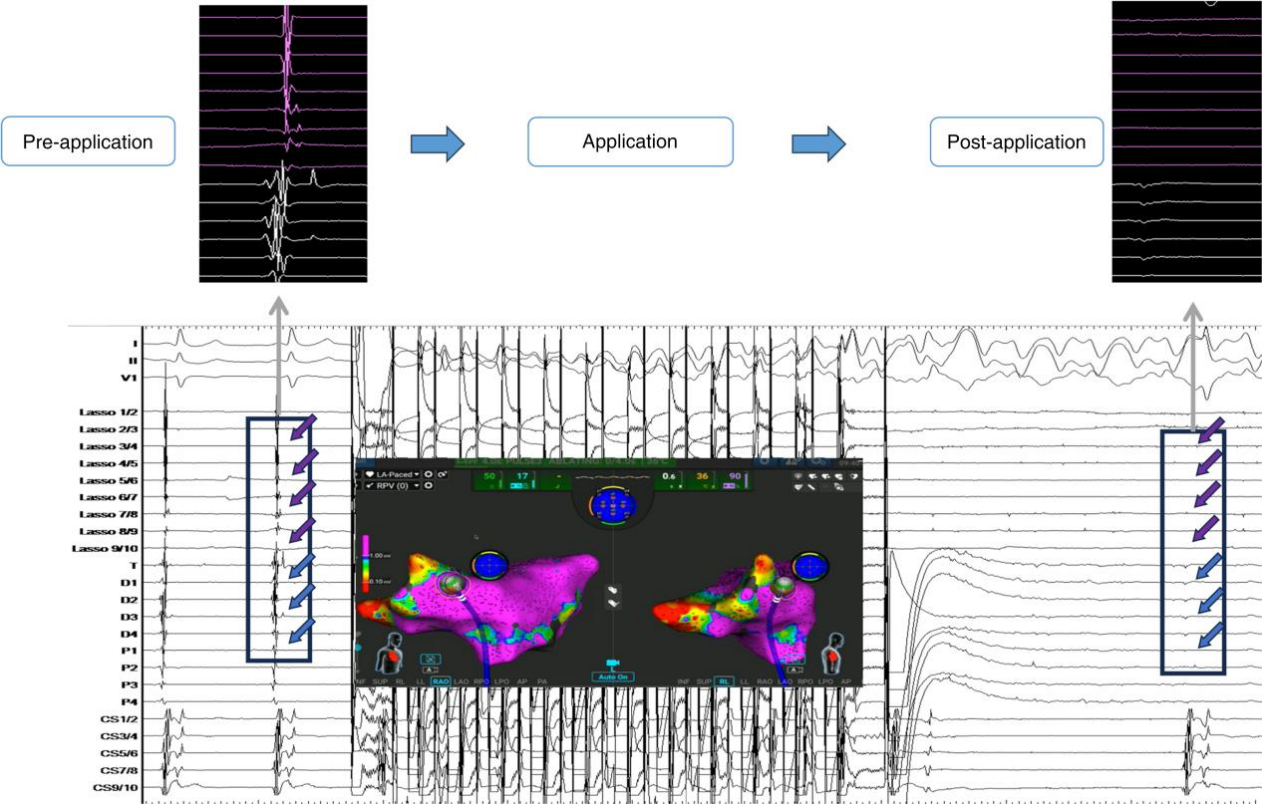
A case of PV isolation with only single application. Pulomonary vein potential disappeared immediately after the first application.

Pulmonary vein isolation has been established as a gold-standard strategy for AF ablation. Thus, investigating the acute procedural success rate and, most importantly, the clinical effectiveness of the present lattice-tip PFA/RFA catheter was imperative. Considering the data of other contemporary thermal modalities,^17,18^ the validated rate of first-pass isolation should be highlighted. Delving further into the specifics, it is noteworthy that the abatement of PV potentials was noted in most PVs after ablating around the PV antrum, approximately halfway or three-quarters around (*Figure 3*). A broader lesion formation with unipolar formed energy delivery and/or existence of a limited amount of conduction fibre in patients with persistent AF is possible. Interestingly, some PVs were isolated using the first couple of applications (*Figure 5*; Supplementary material online, *Video S2*). This phenomenon indicated a potential possible mechanism such as ‘tissue electrical stunning effect.’^19^ This aspect might confound the operators by questioning the PVI durability. The data of the current wave form has been limited to an invasive remapping study 3 months after the procedure irrespective of symptom in a clinical trial (97% per PV, 90% per patient).^3^ As for the long-term durability of PVI in the chronic phase, large-scale reports are anticipated. Initially, as part of real-world data collection, evaluating data from repeat ablation for recurrent atrial tachyarrhythmia is deemed necessary.

### Linear ablation with combination of PFA/radiofrequency ablation application

In persistent AF, PVI alone yields inferior treatment outcomes compared to paroxysmal AF. Although empirical linear ablation as a substrate modification beyond PVI has been explored in prospective randomized trials for persistent AF, no clinical benefit could be concluded.^8–11^ As a contributing factor, the failure to establish a high acute procedural success rate with previous thermal ablation techniques has been considered. Nevertheless, Reddy *et al.*^3^ showed a satisfactory procedural data in linear ablations (100% of acute success and 91% of the 3-month lesion durability) which holds the potential of becoming a viable treatment option for a more challenging AF. Moreover, recurrent tachyarrhythmia as AT could be prevented.^20^ The remarkable acute procedural data on linear ablation confirmed in the present study could have contributed to significantly addressing limitations in linear ablation. Furthermore, considering the reported risk of coronary artery spasm during PFA at the posterior mitral isthmus,^21,22^ the anterior mitral isthmus line could serve as a considerable alternative to mitigate this risk. Pulsed-field ablation was utilized near right superior pulmonary vein to prevent unexpected injury of the phrenic nerve via the RFA according to the manufacturer’s recommendation. Conversely, RFA was utilized for the anterior part of the LA in the previous first-in-human trial.^2^ We followed and combined this strategy aiming for a more contiguous lesion formation.^23^ Alternating the advantages of each energy source indicates an optimized strategy for achieving anterior mitral isthmus block without compromising safety.

### Safety profile

The PFA concept originates from the energy delivery with myocardium-specificity. Hence, complications representative of thermal ablation, such as oesophageal injury and PV stenosis are unlikely to occur, which has been confirmed in previous studies using PFA devices including the lattice-tip PFA/RFA catheter.^24,25^ In this study, neither oesophageal and pulmonary symptoms was clinically observed. None of the other clinical complications occurred, which indicated an excellent safety profile of this system.

### Effectiveness

Pulsed-field ablation has been reported to injure the tissue with the absence of early proarrhythmic effect (i.e. inflammation) or delayed lesion development.^26,27^ Adjacent intrinsic autonomic nervous system is likely to be preserved.^28^ These theories indicated a different clinical time course of the PFA from other thermal ablations. Thus, defining the blanking period as 90 days after PFA including other thermal ablations remains unclear. Although data on the blanking period after PFA remain limited,^29,30^ in this study, the acute clinical efficacy seemed feasible considering that 15% presented early recurrence.

### Clinical implication

The present study collated a single-centre real-world initial data after commercialization of the Affera PFA/RFA system. The unique concept of this system is through the combination of the capability of toggling the PFA and RFA energy delivery, integration to three-dimensional mapping system, and allowing a point-by-point strategy, which offered a remarkable acute success rate in PVI and linear ablations without compromising the safety profile under deep sedation. Of note, we underscored on the indication on patients with persistent AF, a cohort potentially benefiting from ablation beyond PVI. This approach is in line with some positive data^12,13^ indicating a clinical benefit in these patients if substrate and/or triggers are identified successfully and treated individually. Hence, the present preliminary data highlight the future perspective in the ablation strategy for persistent AF. Further studies should explore the mid- and long-term clinical course.

### Limitations

This study has several limitations. First, the present analysis is limited by the single-arm clinical study design and the small sample size. Furthermore, the cohort was limited to patients with persistent AF. Second, no waiting period to evaluate PV conduction recurrence was completed in patients in which only PVI was conducted. However, in these patients, provocative manoeuvres using adenosine test were conducted. Further studies should be conducted regarding the need for additional bonus applications. Third, this study was not designed to evaluate chronic efficacy. Follow-up was mainly performed via telephone interview without an implantable monitoring recorder, thus, asymptomatic recurrence could be underestimated. Loss to follow-up was also notable. To analyse both efficacy and safety, mid- and long-term clinical courses need to be carefully assessed.

## Conclusions

This initial real-world data regarding AF ablation using the lattice-tip PFA/RFA ablation system confirmed a remarkable procedural efficacy of this system including the safety profile. Reproducibility and clinical benefit should be explored with further pivotal studies including the mid- and long-term clinical course.

## Supplementary Material

euae129_Supplementary_Data

## Data Availability

The data that support the findings of this study are available on request from the corresponding author.
